# Transcription fidelity and its roles in the cell

**DOI:** 10.1016/j.mib.2017.08.004

**Published:** 2018-04

**Authors:** Pamela Gamba, Nikolay Zenkin

**Affiliations:** Centre for Bacterial Cell Biology, Institute for Cell and Molecular Biosciences, Newcastle University, Baddiley-Clark Building, Richardson Road, Newcastle Upon Tyne NE2 4AX, UK

## Abstract

Accuracy of transcription is essential for productive gene expression, and the past decade has brought new understanding of the mechanisms ensuring transcription fidelity. The discovery of a new catalytic domain, the Trigger Loop, revealed that RNA polymerase can actively choose the correct substrates. Also, the intrinsic proofreading activity was found to proceed via a ribozyme-like mechanism, whereby the erroneous nucleoside triphosphate (NTP) helps its own excision. Factor-assisted proofreading was shown to proceed through an exchange of active centres, a unique phenomenon among proteinaceous enzymes. Furthermore, most recent *in vivo* studies have revised the roles of transcription accuracy and proofreading factors, as not only required for production of errorless RNAs, but also for prevention of frequent misincorporation-induced pausing that may cause conflicts with fellow RNA polymerases and the replication machinery.

## Highlights

• The Trigger Loop is one of the major determinants of transcription fidelity.
• Intrinsic proofreading occurs via transcript-assisted cleavage.
• Factor-assisted proofreading takes place via exchange of RNAP active centres.
• Misincorporation is a major source of transcription pausing.
• Another role of fidelity is the prevention of conflicts with other cellular processes.

**Current Opinion in Microbiology** 2018, **42**:13–18

This review comes from a themed issue on **Cell regulation**

Edited by **Rita Tamayo** and **Jan-Willem Veening**

For a complete overview see the Issue and the Editorial

Available online 29th September 2017

**http://dx.doi.org/10.1016/j.mib.2017.08.004**

1369-5274/© 2017 The Authors. Published by Elsevier Ltd. This is an open access article under the CC BY license (http://creativecommons.org/licenses/by/4.0/)..

## Introduction

Gene expression relies on the accurate copy of genetic information. The fidelity of RNA synthesis results from the accuracy of correct NTP selection (versus non-complementary NTPs and complementary 2'-deoxy NTPs), the proofreading of misincorporation events, and the efficiency of extension of the misincorporated nucleotide. In this review, we summarize the structural and biochemical determinants of transcription fidelity that have been uncovered in the last decade, and we describe very recent insights on the consequences that stalled misincorporated complexes may have on cellular functions and gene expression.

## Determinants of the accuracy of NTP choice

**Figure 1:**
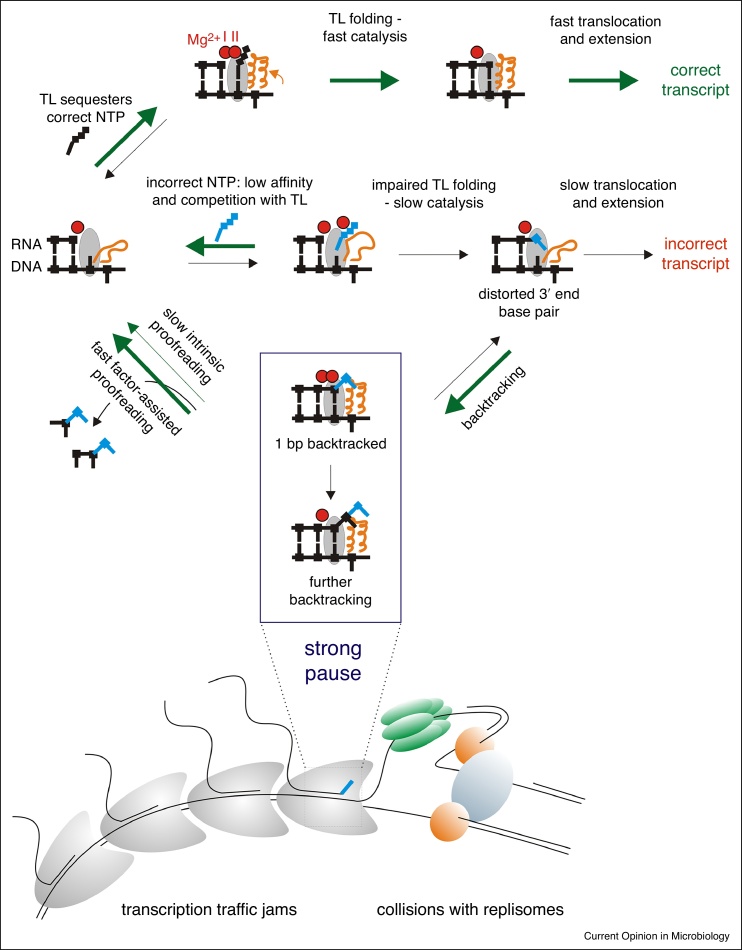
Multistep processes ensuring transcription fidelity. A schematic representation of the active centre of RNAP is given for different transcription intermediates, and shows template DNA and RNA (black lines), metal ions (red circles), the *i* + 1 site (grey oval) and the Trigger Loop (orange ribbon). Correct and incorrect incoming NTPs are coloured in black and blue, respectively. Green arrows show the direction of reactions leading to a correct transcript. The different thickness of the arrows serves only as a qualitative indication of the rates of reactions or conformational changes. At the bottom of the figure, a cartoon depicts a stalled misincorporated elongation complex, which may potentially cause transcription traffic jams with trailing RNAPs (left), and conflicts with replication forks (right).

For a long time, the catalysis of phosphodiester bond formation by RNA polymerase (RNAP) was thought to be performed solely via a two metal ion (Mg^2+^) mechanism within a relatively rigid active centre. However, at saturating NTPs concentrations (close to cellular levels), such a ‘motionless’ active site would provide as low as ~10-fold kinetic discrimination against some non-complementary NTPs (though 10^3^ for certain misincorporations), and would not discriminate at all against complementary 2'-deoxy NTPs [1^••^]. The discovery of a flexible domain of the active site, the Trigger Loop (TL) [2^••^], revealed that the active centre of RNAP actively participates in choosing NTPs via an induced fit mechanism [1••, 3]. TL is essential for the catalysis of phosphodiester bond formation, and it acts by stabilising the transition state of the reaction [1••, 4]. The key property of the TL for the accuracy of transcription is its ability to accommodate catalytically active (folded) and inactive (open) structural states. The correct NTP binding in the *i* + 1 site (grey in Figure 1) induces folding of the TL (orange in Figure 1), which, in turn, participates in the catalysis of nucleoside monophosphate (NMP) incorporation into the transcript. Binding of a non-cognate NTP in the *i* + 1 site cannot induce productive folding of the TL because of the wrong geometry of base pairing with the template (in case of non-complementary NTPs) or the lack of critical contacts of the NTP's sugar moiety with the TL (in case of complementary deoxy NTPs) [1^••^]. Such an induced fit mechanism of selection provides 1–3 extra orders of magnitude of kinetic discrimination against non-complementary NTPs, and 3 orders of magnitude against complementary dNTPs [1^••^].

The affinity discrimination against non-complementary NTPs takes place due to their weaker base pairing with the template, and may increase discrimination by more than an order of magnitude. Furthermore, the TL competes with non-cognate NTPs in the *i* + 1 site [1^••^], while sequestering the correct NTPs bound there [5]. Such ‘active’ expulsion of only wrong substrates adds another order of magnitude to the discrimination against non-complementary NTPs. Notably, TL-mediated expulsion is the only ‘affinity’ component for discrimination against dNTPs because the affinity of their binding in the active site is the same as for ribonucleotides [1^••^].

It must be noted that, while the above-mentioned mechanisms are general and conserved, their efficiencies may vary greatly depending on the identity of incoming NTP, the acceptor base in the template DNA as well as surrounding sequences [1••, 6, 7]. For example, overall kinetic discrimination in the active centre fluctuates from 10^3^ to 10^5^ fold, depending on the particular misincorporation [1^••^]. The lower affinity of non-complementary NTPs may improve discrimination to 10^5^–10^7^ fold, although this may differentially drop according to the concentrations of NTPs in the cell. It should also be noted that some accessory factor may influence RNAP accuracy, such as, in *E. coli*, the global transcription regulator DksA, that binds close to the RNAP active centre and slows down the incorporation of erroneous nucleotides [8^•^].

## The fate of misincorporated complexes

Misincorporation does happen occasionally. Because of the absence of Watson-Crick base pairing with the template, the RNA 3' end becomes misaligned in various ways, relatively to the rest of RNAP active centre. This impairs the catalysis of the subsequent NMP addition, though to various extents depending on the mismatched pair at the 3' end of RNA [6], the incoming NTP and surrounding sequences. Thermodynamically, however, misincorporated complexes are likely to accommodate a 1 base pair (bp) backtracked state [9^••^]. In this conformation, the erroneous NMP of the 3' end loses contacts with the template and flips out of the active site, thus shifting the elongation complex by 1 bp backwards (Figure 1). Backtracking of these complexes may continue even further, depending on the thermodynamics of surrounding sequences (Figure 1). Backtracked complexes are inactive in transcript elongation because the 3' end of RNA is away from the active site. Only an occasional reversion of backtracking, followed by the slow extension of the incorrect 3' end, would result in the retention of the misincorporated nucleotide in the transcript. These delays are one of the major contributors to the overall fidelity of synthesis of the final RNA products as they provide time for resolution of misincorporated complexes via proofreading mechanisms. However, at the same time, they also constitute a major source of paused complexes in the cell, as we discuss below.

## Intrinsic proofreading of transcription

RNAP active centre is able to hydrolyse the phosphodiester bonds of the transcript [10^•^]. This reaction is used by RNAP to proofread the mistakes in RNA, as the new 3' end of RNA generated as a result of hydrolysis becomes available for extension (Figure 1). The reaction is catalysed by the same two metal ions mentioned earlier and the TL [11], though the extent of the TL involvement may differ in different organisms [4, 11, 12, 13] In the 1 bp backtracked state, adopted after misincorporation, it is the second phosphodiester bond that is positioned in the active site for hydrolysis (Figure 1). Interestingly, in this conformation the erroneous 3' end NMP of the transcript directly participates in the hydrolysis, thus facilitating its own removal in the form of a dinucleotide [9^••^]. The 3' end NMP provides coordination bonds for the second catalytic metal ion, as well as stabilises and activates the attacking water molecule [9^••^]. Though it is difficult to assess the contribution of this transcript-assisted proofreading to the overall fidelity of transcription, *in vitro* it was shown to proofread most misincorporation events before the wrong transcript is extended, even in high concentrations of substrates [9^••^].

## Factor-assisted proofreading of transcription

Most organisms possess factors that strongly stimulate hydrolysis of the phosphodiester bonds in the transcript and thus proofreading of transcription. In bacteria these are the Gre factors, while archaea and eukaryotes employ homologues of RNA polymerase II factor TFIIS. These accessory factors stabilise the second catalytic metal ion and activate the attacking water molecule [14•, 15]. To do that, they physically displace and substitute for the TL in the RNAP active centre, thus changing the catalytic properties of RNAP from slow intrinsic hydrolysis (catalysed by TL) to fast factor-assisted hydrolysis [16••, 17••]. *In vitro*, *Thermus aquaticus* GreA stays bound to the elongation complex, but is inactive during correct synthesis, and substitutes for the TL only upon misincorporation or occasional backtracking [16^••^]. *E. coli* GreB, however, was shown to dissociate quickly from the elongation complex, reflecting possible different modes of regulation of the activities of different Gre factors [18]. *In vitro*, GreA proofreads almost all misincorporation events before their extension [9^••^], but the general contribution of Gre to prevent retention of mistakes in the final transcripts could be moderate [7, 19••, 20•].

## Visualizing transcription errors *in vivo*

In bacteria, the study of transcriptional fidelity *in vivo* relied for a long time on *lacZ* reporter genes carrying a nonsense codon in the open reading frame [21, 22, 23]. Such constructs allowed to estimate transcriptional error rates of ~10^-5^–10^-4^ [21, 22], and were used to identify RNAP mutants with reduced accuracy of chain elongation [23]. More recently, similar constructs have detected an increase in error rate in a *greA* mutant of *Streptococcus pneumoniae* [20^•^], and in a *dksA* mutant of *E. coli* [8•, 24]. Comparable approaches in *Saccharomyces cerevisiae* gave contradictory results on the role of TFIIS [25, 26, 27].

A new reporter assay, based on the suppression of a missense mutation in the active site of Cre recombinase, has recently been developed for the detection of G?A (misincorporation of A instead of G) errors [28, 29]. In *E. coli*, a *greA* mutant strain showed over 100-fold increase in error rate, similarly to a double *greA greB* mutant, while deletion of *greB* alone did not have any effect, revealing a major role for GreA in transcription proofreading [28]. Overexpression of GreB could however complement deletion of *greA* [28]. In yeast, the same approach successfully detected an increase in G?A errors in strains lacking TFIIS or the RNA polymerase II subunit Rpb9, with the former inducing 3 times more errors than the latter, and was used to identify new fidelity mutants of Pol II, which mapped in the Trigger Loop, the bridge helix, and in the sites involved in binding to TFIIS [29].

In recent years, next-generation sequencing technologies have allowed the study of transcription fidelity in greater detail [7, 19••, 20•, 30••, 31]. Nascent elongating transcript sequencing (NET-seq) selectively captures the 3' end of transcripts that are being actively elongated by the RNAP, and has revealed sequence-dependent transcriptional pausing with nucleotide resolution [32, 33••]. When applied to the analysis of errors in the actively transcribing complexes, it revealed that misincorporated complexes are 1–3% of all elongation complexes in wild-type cells of *Saccharomyces cerevisiae* and *E. coli*, respectively [19^••^], a much higher proportion than expected from the overall error rate of RNA synthesis. In the absence of cleavage factors (TFIIS or Gre), the fraction became 7% and 5%, respectively [19^••^]. A somewhat lower proportion of misincorporated complexes was observed in another study [30^••^], though the native RNA preparation protocol used in that case may have favoured the intrinsic proofreading activity of RNAP, as we have discussed previously [19^••^].

The misincorporation pattern showed a strong bias towards G?A misincorporation [19••, 30••], in line with previous *in vitro* observations [1••, 6, 7], and data suggested that CG motifs increase G?A misincorporation [30^••^]. This bias however seems to be apparent only at positions of very frequent misincorporation (hotspots), which are a minor fraction of the total events [19^••^]. Interestingly, in *E. coli* these hotspots are ~8 times more abundant in untranslated regions compared to protein coding sequences, while no difference was observed in *S. cerevisiae* [19^••^].

## Phenotypic consequences of transcription infidelity

The study of transcription fidelity *in vivo* remains challenging, but several reports have linked transcription errors to detrimental cellular phenotypes in eukaryotes [34, 35, 36, 37, 38].

In bacteria, transcriptional infidelity was shown to be a significant source of molecular noise, which could lead to heritable phenotypic changes via activation of a bistable switch [39••, 40]. Bistable feedback loops regulate important pathways in bacteria, including cellular differentiation, virulence and expression of metabolic genes, and are particularly sensitive to noise in gene expression [41]. In *E. coli*, deletion of both *greA* and *greB*, but not single deletions alone, considerably increased the switching frequency of the *lac* operon [39••, 40], and the error-prone *ack-1* mutation of RNAP also promoted the switching [39^••^].

It seems now questionable whether transcription infidelity influences cellular phenotypes via the actual production of erroneous proteins. Misincorporation events cause long-lived pauses *in vitro* because of backtracking [9••, 42]. Backtracked pauses were shown to cause conflicts with replication forks *in vivo*, leading to detrimental consequences such as double strand brakes and genome instability [43••, 44•]. It was also suggested that queues of RNAPs forming behind the stalled one might actually be the main obstacle to replication fork progression and/or cause changes in gene expression [20^•^]. The substantial proportion of misincorporated complexes detected by NET-seq indicates that such stalled complexes are slowly resolved *in vivo*, and therefore may be a major source of conflicts with fellow RNAPs and replication complexes [19^••^]. In this context, the physical block of transcription of regulatory genes is likely to have a greater impact on molecular noise than the rare mistakes in final RNA products. Also, accumulation of misincorporated complexes may exacerbate the conflicts between RNAP and other cellular machineries, which could be responsible for the deleterious phenotypes that have been linked to infidelity.

Consequently, the most relevant role of cleavage factors Gre and TFIIS (and its homologues) *in vivo* may be the resolution of stalled misincorporated complexes [19••, 20•]. Gre factors and DksA were previously shown to be important to resolve conflicts between DNA replication and transcription under certain conditions [44•, 45]. For instance, viability of *E. coli* strains lacking *greA* and *dksA* is reduced when DNA repair is compromised [44^•^]. Also, DksA was shown to ensure replication completion upon amino acid starvation by removing transcription roadblocks [45]. Furthermore, a triple mutant *greA greB dksA* grows extremely slowly and with a high degree of filamentation [46, 47] and showed a significant decrease in replication fork progression [45]. Severe growth and morphological defects, including aberrant nucleoid morphology, were also observed in a *greA* mutant of *S. pneumoniae*, which does not encode other cleavage factors nor DksA homologues [20^•^].

## Conclusions

Recent biochemical, genetic and next-generation sequencing advances have revised and improved our view of the mechanisms and the roles of transcription fidelity in both bacteria and eukaryotes. However, a number of questions remain unanswered. For example, the exact structural basis for the differences in discrimination against various misincorporation events remains only hypothetical. Also, the involvement of transcription factors such as DksA, or RNA polymerase II subunits such as Rbp9, in transcription accuracy is still unclear. Most interestingly, the mechanisms by which cells resolve the apparently detrimental misincorporated complexes in the absence of proofreading factors remain elusive.

## References and recommended reading

Papers of particular interest, published within the period of review, have been highlighted as:

• of special interest
•• of outstanding interest
